# Spatial regulation of plant hormone action

**DOI:** 10.1093/jxb/erad244

**Published:** 2023-07-04

**Authors:** Cynthia Wong, David Alabadí, Miguel A Blázquez

**Affiliations:** Instituto de Biología Molecular y Celular de Plantas (CSIC-UPV), 46022-Valencia, Spain; Instituto de Biología Molecular y Celular de Plantas (CSIC-UPV), 46022-Valencia, Spain; Instituto de Biología Molecular y Celular de Plantas (CSIC-UPV), 46022-Valencia, Spain; Universidad de Sevilla, Spain

**Keywords:** Metabolism, plant hormones, plant hormone sensor, signaling, spatial regulation, transport

## Abstract

Although many plant cell types are capable of producing hormones, and plant hormones can in most cases act in the same cells in which they are produced, they also act as signaling molecules that coordinate physiological responses between different parts of the plant, indicating that their action is subject to spatial regulation. Numerous publications have reported that all levels of plant hormonal pathways, namely metabolism, transport, and perception/signal transduction, can help determine the spatial ranges of hormone action. For example, polar auxin transport or localized auxin biosynthesis contribute to creating a differential hormone accumulation across tissues that is instrumental for specific growth and developmental responses. On the other hand, tissue specificity of cytokinin actions has been proposed to be regulated by mechanisms operating at the signaling stages. Here, we review and discuss current knowledge about the contribution of the three levels mentioned above in providing spatial specificity to plant hormone action. We also explore how new technological developments, such as plant hormone sensors based on FRET (fluorescence resonance energy transfer) or single-cell RNA-seq, can provide an unprecedented level of resolution in defining the spatial domains of plant hormone action and its dynamics.

## Teaser

We present the basic principles of what we consider to be the most important mechanisms, namely metabolism, transport, and perception/signal transduction, contributing spatial specificity to plant hormone action.

## Introduction

Plant hormones are small signaling molecules of diverse chemical nature that regulate almost every aspect of a plant’s life, from developmental decisions to stress responses and acclimation (Kamiya, 2010). In most cases, plant hormones typically exert their roles through signaling pathways that eventually affect gene expression, although they are also known to regulate other aspects of cellular homeostasis, such as ion fluxes, cytoskeleton dynamics, or vesicle trafficking (MacRobbie, 2000; Locascio *et al.*, 2013; Chen *et al.*, 2014; Xu *et al.*, 2014). In this context, recent research has shown that signaling elements of several plant hormone pathways exert their activity in biocondensates in the cytoplasm (Emenecker *et al.*, 2020). NONEXPRESSOR OF PATHOGENESIS-RELATED GENES1 (NPR1) forms biocondensates in response to salicylic acid to suppress pathogen-triggered programmed cell death (Zavaliev *et al.*, 2020). Similarly, the transcription factors AUXIN RESPONSE FACTOR7 (ARF7) and ARF19 form biocondensates in the cytoplasm to limit auxin responsiveness in non-elongating tissues of the root (Powers *et al.*, 2019). One paradigmatic difference between plant and animal hormones is that the latter are synthesized in special endocrine glands and must be transported to their target organs through the blood stream, while plants lack dedicated glands for hormone synthesis. In principle, every plant cell has the ability to synthesize different hormones and to perceive hormones and respond accordingly, as shown for instance by the distinct response patterns elicited by different hormone treatments in cultured plant cells (MacRobbie, 2000). However, there is ample evidence that plant hormone action is subject to spatial regulation. For example, application of a plant hormone causes very different biological responses in different organs, supported by qualitatively different transcriptomic changes in each organ (Cao *et al.*, 2006; Paponov *et al.*, 2008; Brenner and Schmülling, 2012), suggesting that the hormone response circuit may be distinctly organ specific in its wiring. In addition, the presence of several plant hormones and related metabolites has been detected in the xylem and phloem sap (Hoad and Bowen, 1968; Zeevaart and Boyer, 1984; Bishopp *et al.*, 2011b), suggesting that the localization of the synthesis and of the action may sometimes be different. One very elegant piece of work that supports the relevance of plant hormone-related metabolites as communicating entities is the identification of the antheridiogen responsible for male sex determination in ferns (Grayson *et al.*, 2014). Antheridiogen is an inactive gibberellin molecule (GA_9_-Me) synthesized by early-maturing prothalli which is then taken up by younger individuals in the colony, where it is modified into bioactive gibberellin and triggers male organ differentiation.

In this review, we will first describe general principles and mechanisms that govern the spatial regulation of plant hormone action, and then focus on a selection of biological processes where several of these mechanisms operate in a coordinated manner to generate spatial information and differential responses.

## General mechanisms for the spatial regulation of plant hormone action

Experimental evidence accumulated over the past 30 years of plant hormone research indicates that there are three main ways for hormones to have a local effect in plants: (i) by ensuring local biosynthesis or local inactivation of the hormone; (ii) by changing the transport and thus the spatial distribution of the hormone; and (iii) by differentially affecting the responsiveness to the hormone in different locations.

### Spatial regulation of plant hormone metabolism

The pool of active plant hormones in specific locations can be controlled at three metabolic levels: (i) hormone biosynthesis; (ii) hormone inactivation, either reversible or irreversible; and (iii) hormone reactivation from inactive pools. To the best of our knowledge, the spatial control at the three metabolic levels is mainly achieved by spatially regulated expression of genes encoding key enzymes.

There are numerous reports in the literature documenting the role of local plant hormone biosynthesis in growth and developmental processes. For example, the differentiall cell growth that leads to apical hook formation in etiolated seedlings is due, in part, to local, asymmetric biosynthesis of ethylene and auxin (Peck *et al.*, 1998; Raz and Ecker, 1999; Stepanova *et al.*, 2007). In pea, the asymmetric distribution of *Pisum sativum ACC OXIDASE1* (*PsACO1*) transcripts across the hook correlates with ACO activity being highest on the concave side, suggesting that ethylene accumulation on this side suppresses cell elongation (Peck *et al.*, 1998). Similarly, *TRYPTOPHAN AMINOTRANSFERASE RELATED2* (*TAR2*), an enzyme in the first step of the auxin biosynthesis pathway, is also expressed in the concave side, contributing to local auxin accumulation (Stepanova *et al.*, 2008). In other cases, the local biosynthesis of the plant hormone could be associated not only with local action, but also with its transport. This is the case with strigolactones and abscisic acid (ABA). The strigolactone biosynthetic genes *MORE AXILLARY BRANCHES1* (*MAX1*), *MAX3*, and *LATERAL BRANCHING OXIDOREDUCTASE1* (*LBO1*) are expressed almost exclusively in the vasculature where they generate the pool of precursors or active strigolactone that is transported (Booker *et al.*, 2005; Liang *et al.*, 2011; Brewer *et al.*, 2016). Similarly, the ABA biosynthetic genes *ALDEHYDE OXIDASE3* (*AAO3*) and *ABA DEFICIENT2* (*ABA2*) are expressed in phloem companion cells synthesizing ABA that is transported to stomata (Kuromori *et al.*, 2014). Spatially regulated hormone inactivation also contributes to the regulation of the ABA pool. Two genes encoding the catabolic ABA 8ʹ-hydroxylase increase their expression at sites of transport—the vasculature—and action—the stomata—to reduce ABA levels in response to high humidity (Okamoto *et al.*, 2009). The concerted action of plant hormone biosynthesis and catabolic genes in the same tissues to control the pool of active hormone is also observed for gibberellins in the root endodermis, the leading tissue for regulating root growth (Barker *et al.*, 2021). Mechanisms that reversibly inactivate the plant hormone also contribute to local responses. For example, inactivation of auxin by the methyltransferase INDOLE-3-ACETIC ACID CARBOXYLMETHYLTRANSFERASE1 (IAMT1) contributes to the regulation of the pool of active auxin in the carpels and hypocotyl endodermis, which are required for pollen tube growth and gravitropism, respectively (Abbas *et al.*, 2018a, b). Similarly, ABA is reversibly inactivated via glycosylation by UGT75B1, preferentially in actively growing tissues and germinating seeds where high ABA activity is deleterious (Chen *et al.*, 2020). Plants use hormone reactivation as a rapid way to restore local active hormone pools. An example of this mechanism is the glucosidase BETA GLUCOSIDASE1 (BG1), which hydrolyzes glucose-conjugated inactive ABA, and is preferentially up-regulated in vascular tissues in response to drought, providing a way to rapidly increase ABA levels at the site of transport (Lee *et al.*, 2006).

### Regulation of plant hormone transport

Plant hormone transport mechanisms have been extensively reviewed (Park *et al.*, 2017; Anfang and Shani, 2021). In summary, four core strategies have been documented: (i) the generation of a positive sink that delivers the plant hormone to cells or cell compartments where it exerts its functions; (ii) the use of a negative sink to remove the plant hormone from its place of action; (iii) cell-to-cell transport; and (iv) long-distance transport through tissues and organs.

As an example of a positive sink for an active plant hormone, GA_3_ and GA_4_ have been shown to be imported into root endodermal cells by the NTR1/PTR FAMILY PROTEIN3 (NPF3) transporter, where they are necessary to promote root elongation (Tal *et al.*, 2016) through the degradation of DELLA proteins (Dill *et al.*, 2001; Úbeda-Tomás *et al.*, 2008). However, sometimes the transported molecule is not the active plant hormone, but a precursor of its synthesis. That is the case for NPF7, which transports indole-3-butyric acid into root cap cells, where it is metabolized into the active indole-3-acetic acid, in order to generate the auxin gradient required for the gravitropic response (Watanabe *et al.*, 2020). Negative sinks can be generated, for instance, by importing the active plant hormone into the cytosol while the corresponding hormone receptors face the outer intercellular space; this mechanism has been reported for cytokinin, which is imported from the apoplast to the cytosol by PURINE PERMEASE14 (PUP14) to modulate morphogenetic events (Zurcher *et al.*, 2016). Similarly, a negative sink of active ABA is generated by importing ABA into mesophyll cells by ABCG17 and ABCG18, where the hormone is inactivated by conjugation; under stress situations, the expression of the transporter decreases, and free ABA is released to promote stomatal closure and other ABA responses (Zhang *et al.*, 2021). The best documented example for cell-to-cell plant hormone transport is the movement of auxin through the influx and efflux carriers whose concerted action causes differential auxin accumulation. For instance, bending of hypocotyls towards a light source depends on redirecting the auxin flux towards the darker side of the organ, caused by the lateral relocalization of the PIN-FORMED3 (PIN3) auxin efflux carrier (Ding *et al.*, 2011). Other plant hormones may also be transported to their site of action via cell-to-cell movement rather than through the xylem/phloem stream. For instance, it has been suggested that strigolactones reach the hypodermal passage cells that support the colonization of micorrhizal fungi in *Petunia axilaris* through the polar localization of an ABCG transporter encoded by *PaPLEIOTROPIC DRUG RESISTANCE1* (*PaPDR1*) (Sasse *et al.*, 2015). In the case of brassinosteroids, the partial complementation of the dwarf phenotype of the brassinosteroid-deficient mutant *constitutive photomorphogenic dwarf* (*cpd*) by local expression of *CDP* in the inner provascular tissue suggests that this plant hormone moves over short distances (Savaldi-Goldstein *et al.*, 2007). However, several pieces of evidence also support the existence of long-distance plant hormone transport. On the one hand, a series of well conducted grafting experiments between mutants in gibberellin biosynthesis has established that the gibberellin precursor GA_12_ is transported from roots to shoots where it is transformed into the active moieties (Regnault *et al.*, 2015); the identity of the translocators involved has not been elucidated yet. On the other hand, the ABCG14 transporter has been implicated in the translocation of root-synthesized cytokinins to the shoot through the vasculature (Ko *et al.*, 2014; Zhang *et al.*, 2014).

### Spatial regulation of plant hormone perception and signaling

Local plant hormone action at the perception or signal transduction level can be achieved by spatially regulating (i) the expression or (ii) the activity of hormone receptors or signaling components. For example, a plant hormone receptor could be expressed only in certain cell types and, in the presence of the hormone, a signaling cascade occurs only in those cell types to activate a particular response. Alternatively, local plant hormone action could be triggered by the presence of a central signaling component in a particular cell type. This is the case for the inhibition of cell division by cytokinins at the root vascular transition zone to balance cell division and differentiation (Dello Ioio *et al.*, 2008). Here, cytokinins reduce auxin sensitivity in this zone of the root to limit cell division by activating expression of the negative regulator of auxin signaling *INDOLE-3-ACETIC ACID INDUCIBLE3* (*IAA3*)/*SHORT HYPOCOTYL2* (*SHY2*). Cytokinin action is directly exerted by the cytokinin response transcription factor ARABIDOPSIS RESPONSE REGULATOR1 (ARR1). Similarly, a signaling component may be active only in certain tissues or cell types. This regulation can be achieved by spatially regulated post-translational modifications of a particular signaling element that affect its activity (see an example in the following sections), or by the presence of a necessary signaling partner in a particular tissue. An example of the latter is the negative regulation of iron uptake by the gibberellin signaling elements DELLA proteins exerted in the root epidermis (Wild *et al.*, 2016). Although DELLAs are also found in other root tissues, this particular regulation is exerted because they interact with and inactivate IRON-DEFICIENCY INDUCED TRANSCRIPTION FACTOR (FIT), an epidermis-specific transcription factor that promotes the expression of genes for iron uptake.

## The spatial regulation of plant hormone pathways in biological contexts

Local plant hormone action is usually the result of a combination of mechanisms involving local metabolism, transport, and local differences in hormone sensitivity. The following is a selection of four situations where the local effect of one or more plant hormones is critical in determining the correct course of action.

### Vascular patterning is determined by two spatially separated fields of auxin and cytokinin action

**Fig. 1.**
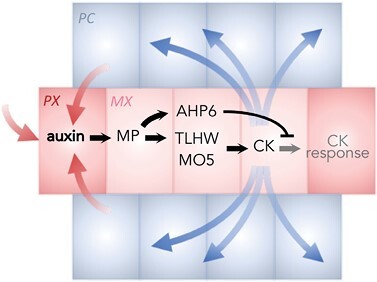
Vascular patterning in the embryonic Arabidopsis root. Proto- and metaxylem identities are established by high levels of auxin and low sensitivity to cytokinins. Procambium identity is associated with high cytokinin levels transported from the xylem, and low auxin levels—facilitated by the activity of PIN auxin efflux carriers. Red color indicates auxin, blue indicates cytokinins. PC, procambium; PX, protoxylem; MX, metaxylem.

The diarch pattern of the vasculature in the primary Arabidopsis root is characterized by a central file of metaxylem cells flanked by two protoxylem cells. This file is surrounded by (pro)cambial cells whose divisions allow radial growth of the roots while maintaining their original organization, and two phloem poles are localized in a perpendicular axis with respect to the metaxylem file (Fig. 1). This pattern is already established during embryogenesis by the specification of cell identities, but its maintenance during post-embryonic growth depends on a self-sustained and robust regulatory mechanism that also provides the plasticity necessary to modify the organization in response to external information. Spatially controlled hormone action plays a crucial role in both phases.

The patterning process is triggered as early as in the globular stage of embryo development, when the precise localization of the two protoxylem cells is established as reference points due to the differential accumulation of auxin being transported from the emerging cotyledons towards the embryonic root through PIN auxin efflux carriers (Friml *et al.*, 2003; Reinhardt *et al.*, 2003). The two cells that receive the highest auxin doses are those located closer to the cotyledons, and this differential accumulation of auxin designates their identity. In those cells, higher auxin levels lead to activation of the DNA-binding ARF5, also known as MONOPTEROS (MP), and a series of downstream targets. Among them, the first one is *TARGET OF MONOPTEROS5* (*TMO5*) (Schlereth *et al.*, 2010; De Rybel *et al.*, 2014), which encodes a basic helix–loop–helix (bHLH) transcription factor whose activity depends on the obligate formation of heterodimers with LONESOME HIGHWAY (LHW) and its paralogs (Ohashi-Ito and Bergmann, 2007; De Rybel *et al.*, 2013). As a result of the TMO5–LHW-dependent transcription of *LONELY GUY4* (*LOG4*), local synthesis of cytokinins is enhanced, and these then permeate to neighboring procambial cells. High cytokinin levels in the procambium are essential for the periclinal cell divisions that increase the number of vascular cell files in the root (Mähönen *et al.*, 2000).

However, the auxin-mediated increase of cytokinin synthesis in the procambium not only promotes divisions of the adjacent cells. High cytokinin levels inhibit protoxylem identity, as shown by the reduced number of procambial cells and ectopic formation of protoxylem in the *wooden leg* (*wol*) mutant, defective in the CYTOKININ RESPONSE1 (CRE1) cytokinin receptor (Mähönen *et al.*, 2000, 2006). How then do xylem cells maintain an active auxin response and the correct identity under high cytokinin levels? The solution is provided, at least in part, through the protoxylem-specific expression of ARABIDOPSIS HISTIDINE PHOSPHOTRANSFER PROTEIN6 (AHP6) (Bishopp *et al.*, 2011a), an inhibitor of cytokinin signaling. This gene is also a direct target of MP/ARF5, so that xylem cells are characterized by high auxin levels and low cytokinin response. In contrast, procambial cells are characterized by high cytokinin levels which promote lateral localization of PIN carriers, increasing the transport of auxin towards xylem cells.

In summary, correct patterning of the vasculature in the root results from the combination of several mechanisms involving spatial regulation of hormone action: (i) transport of auxin from a distant location; (ii) local activation of cytokinin synthesis; (iii) transport of cytokinin towards neighboring cells; (iv) cell type-specific inhibition of cytokinin signaling; and (v) relocation of auxin from procambial to xylem cells. Mathematical modeling using these basic principles has demonstrated that such a regulatory network can generate two mutually exclusive fields of action between auxin and cytokinin that can be maintained even under fluctuating environments that affect growth of the vascular tissue (De Rybel *et al.*, 2014; Muraro *et al.*, 2014).

### The spatial interplay of plant hormone pathways regulates shoot apical meristem function

#### Local action of cytokinins regulate shoot apical meristem function

The shoot apical meristem (SAM) is the source of the cells from which all the above-ground organs of the plant arise. It is spatially divided into different functional domains (Gaillochet and Lohmann, 2015). The most apical, central zone of the SAM contains the stem cell population, which consists of slowly dividing cells. Stem cell identity is maintained by a regulatory loop formed by the transcription factor WUSCHEL (WUS) and the CLAVATA (CLV) signaling module. *WUS* is expressed in a group of cells below the central zone, called the organizing center. Stem cells increase their proliferation rate when displaced from the central zone toward the flanks or the peripheral zone where organ primordia are formed. Both the activity of the SAM and the initiation and activity of organ primordia require local, spatially regulated activity of the hormones cytokinins, auxins, and gibberellins at the metabolic, transport, and signaling levels.

**Fig. 2.**
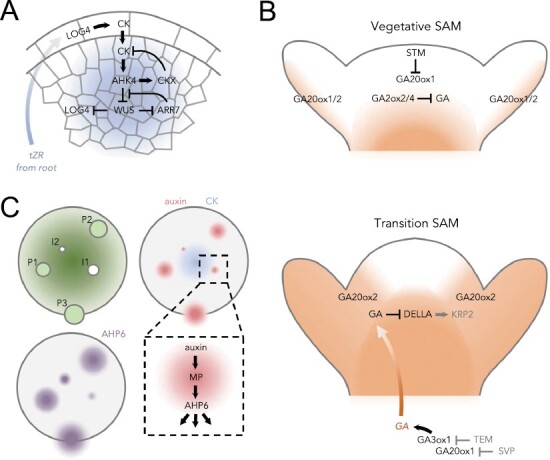
Regulation of SAM functions by local hormone action in Arabidopsis. (A) Maintenance of meristem identity by local production of active cytokinins from precursors transported from roots. (B) The role of gibberellins during the transition to flowering. Gibberellins are maintained outside the SAM during the vegetative phase, but at the onset of flowering there is a transient increase to support a change in the SAM to accommodate new organs, which is due to long-distance transport and local synthesis at the flanks of the meristem. (C) Distinct domains of auxin and cytokinin responses generate a robust phyllotactic pattern for the emergence of new organ primordia in a regular manner. Red color, auxin; blue, cytokinin; purple, AHP6 expression domains. P1, P2, and P3 are previously formed primordia; I1 and I2 are the site where the next primordia will appear and the incipient primordia, respectively.

The sustained activity of WUS and the correct location of *WUS*-expressing cells is of paramount importance for the functioning of the SAM, and here cytokinins play a key role (Fig. 2A) (Leibfried *et al.*, 2005; Chickarmane *et al.*, 2012; Osugi *et al.*, 2017; Landrein *et al.*, 2018). In aerial organs, cytokinins are synthesized via a two-step pathway involving the consecutive action of ISOPENTENYLTRANSFERASE (IPT) and LOG enzymes (Schaller *et al.*, 2015). Of these enzymes, only LOG4 is expressed at the SAM (Miyawaki *et al.*, 2004; Schmid *et al.*, 2005; Yanai *et al.*, 2005). Therefore, cytokinin biosynthesis in the SAM requires import of precursors, such as the LOG substrates, from other tissues, mainly *trans*-zeatin riboside (tZR) synthesized in the root and loaded into xylem vessels by ABCG14 (Ko *et al.*, 2014; Zhang *et al.*, 2014; Osugi *et al.*, 2017; Landrein *et al.*, 2018). LOG4 accumulates exclusively in the epidermal layer of the SAM (Chickarmane *et al.*, 2012). Cytokinins, however, would act in internal tissues to promote the expression of *WUS* in the correct place (Gordon *et al.*, 2009). In concert, they diffuse into the inner layers where they activate the receptor AHK4, with hormone activity being highest where *WUS* is expressed (Chickarmane *et al.*, 2012). Cytokinin levels in the *WUS*-expressing domain are further adjusted by degradation via CYTOKININ OXIDASE3 (CKX3) and CKX5 cytokinin oxidases (Bartrina *et al.*, 2011). WUS, in turn, regulates cytokinin actvity at two levels. On the one hand, it contributes to the maintenance of a high cytokinin response by directly repressing the expression of several negative regulators of cytokinin signaling (Leibfried *et al.*, 2005). On the other hand, WUS reduces cytokinin biosynthesis by repressing the expression of *LOG4* (Chickarmane *et al.*, 2012). Although auxin activity is highest at the flanks of the SAM (see below), auxin helps to maintain high cytokinin activity in the organizing center through ARF5-mediated reduction of the expression of several negative regulators of cytokinin signaling (Zhao *et al.*, 2010). In summary, local cross-regulation between the WUS–CLV module and cytokinins determines the location and activity of WUS and thus the size of the SAM. Consistent with this, *abcg14* or *log* mutants have SAM and inflorescences of reduced size (Kurakawa *et al.*, 2007; Kuroha *et al.*, 2009; Ko *et al.*, 2014; Zhang *et al.*, 2014; Osugi *et al.*, 2017), while the opposite phenotype is observed in *ckx3,5* mutants that overaccumulate cytokinins (Bartrina *et al.*, 2011).

Interestingly, plants use the tZR precursor as a systemic signal to convey information on nutrient availability and regulate SAM activity. In particular, biosynthesis and transport of tZR are enhanced by nitrate in soil, and at least partly by up-regulating the expression of *IPT3* and *IPT5* genes, thus connecting the organogenesis capacity of the SAM with nutrient availability (Landrein *et al.*, 2018). In this manner, there is a positive correlation between the level of nutrients in the soil, the cytokinin activity in the SAM, and the size of the SAM.

#### Spatial regulation of gibberellin metabolism at the shoot apical meristem

Gibberellins have opposing effects on SAM activity during the vegetative phase and the transition to flowering, achieved by spatial regulation of gibberellin metabolic genes (Fig. 2B). In contrast to the requirement for local cytokinin activity for SAM function, gibberellins are actively excluded from the SAM during the vegetative phase to maintain cells in the undifferentiated state (Hay *et al.*, 2002; Jasinski *et al.*, 2005). This is achieved by spatial regulation of gibberellin metabolism at two levels (Fig. 2B; upper panel). On the one hand, gibberellin biosynthesis is prevented in the SAM by SHOOT MERISTEMLESS (STM), a KNOX transcription factor that confers meristem identity and that represses the expression of the *AtGA20ox1* gene, which encodes an enzyme catalyzing a limiting step in gibberellin biosynthesis (Hay *et al.*, 2002). On the other hand, this effect is enhanced by preventing access of active gibberellins derived from other organs to the SAM by local expression of genes encoding the gibberellin-deactivating enzymes AtGA2ox2 and AtGA2ox4 in its basal region (Jasinski *et al.*, 2005).

During the transition to flowering under inductive photoperiods, the SAM transiently increases in size, acquiring the shape of a dome to accommodate flower promordia (Kinoshita *et al.*, 2020), coinciding with an increase of gibberellin content in the shoot apex (Eriksson *et al.*, 2006). The increase in gibberellins responds to the simultaneous, differential spatial regulation in the SAM of two genes involved in gibberellin metabolism (Fig. 2B; lower panel) (Kinoshita *et al.*, 2020). *AtGA20ox2* expression is transiently induced in the peripheral zone of the SAM, while that of the gibberellin catabolic gene *AtGA2ox4* is reduced. Consistently, the *ga20ox2* mutant showed reduced meristem size during transition, while the *ga2ox4* SAM behaved like the wild type. The importance of gibberellins in promoting the enlargement of the SAM during the transition to flowering is also evidenced by the fact that forced, local reduction of gibberellins or gibberellin activity in the SAM by the expression of *AtGA2ox7* or a hyperactive DELLA protein, respectively, under the promoter of the KNOX gene *KNOTTED-LIKE FROM ARABIDOPSIS THALIANA1* (*KNAT1*) also causes a reduction in the size of the SAM and a delay in flowering (Galvao *et al.*, 2012; Porri *et al.*, 2012; Kinoshita *et al.*, 2020).

Spatial regulation of gibberellin metabolic genes in the region below the SAM by floral repressors also contributes to regulate the flow of gibberellins toward the shoot apex during the transition to flowering (Fig. 2B; lower panel). For instance, inactivating the floral repressors TEMPRANILLO (TEM) and SHORT VEGETATIVE PHASE (SVP) releases the repression that they exert on the gibberellin biosynthetic genes *AtGA3ox1* and *AtGA20ox1* at the base of the SAM, respectively, thus allowing gibberellin production in the shoot apex required for the floral transition (Osnato *et al.*, 2012; Andrés *et al.*, 2014).

Induction of flowering involves elongation of the stem, and the SAM, especially the rib meristem, helps to form new cells. Although it is not known how gibberellins reach the rib meristem, they promote cell division by degrading DELLA proteins that would otherwise up-regulate expression of the cell cycle inhibitor *KIP-RELATED PROTEIN2* (*KRP2*) (Fig. 2B; lower panel) (Serrano-Mislata *et al.*, 2017). Deactivating gibberellins in the SAM through the expression of *pKNAT1:AtGA2ox7* also causes a strong reduction in stem elongation (Porri *et al.*, 2012), probably related to the action of DELLAs in the rib meristem, where it is also expressed *KNAT1*.

#### Local action of auxins and cytokinins determines the position of organ primordia

As meristematic cells are displaced from the central to the peripheral zone of the SAM, they enter into a differentiation process that leads to the formation of organ primordia (i.e. leaves and flowers). New organ primordia are arranged into a distinctive spatiotemporal pattern in the SAM referred to as phyllotaxis (Reinhardt *et al.*, 2003). Phyllotaxis is controlled primarily by auxins, with cytokinins downstream providing robustness of organ arrangement (Reinhardt *et al.*, 2003; Besnard *et al.*, 2014). This process involves spatial regulation of hormone pathways at the metabolic, transport, and signaling level.

The appearance of an organ primordium at the flank of the SAM is marked by an auxin maximum driven by auxin transport via PIN1 and influx carriers through the epidermis (Benkova *et al.*, 2003; Reinhardt *et al.*, 2003; Kierzkowski *et al.*, 2013). The primordium becomes an auxin sink that withdraws auxin from the surroundings (Fig. 2C). This creates an ‘inhibitory field’ with gradually reduced auxin levels that prevents the formation of a new auxin maximum nearby. Only when the effect of the sink diminishes, because the distance to the sink is too great, can a new auxin maximum be formed (Reinhardt *et al.*, 2003). In fact, local aplication of auxin to the flank of the shoot apex of a *pin1* mutant to artificially create an auxin maximum is enough to trigger the formation of a flower primordium (Reinhardt *et al.*, 2003).

Importantly, mechanical tensions generated at the SAM due to the growth of organ primordia contribute to the coordinated polar localization of the PIN1 transporter in neighboring cells toward the auxin sink (Jönsson *et al.*, 2010; Nakayama *et al.*, 2012). The cell wall loosening triggered by auxin in one cell causes stress in the cell wall of the neighboring cell, which promotes localization of PIN1 in the plasma membrane of that side of the cell (Jönsson *et al.*, 2010). The higher the tension at the plasma membrane due to polarized stress at the cell wall, the higher the PIN1 accumulation, directing auxin flux toward the auxin sink (Nakayama *et al.*, 2012).

Although auxin transport is the primary force marking the sites of organ primordia, spatially regulated auxin biosynthesis and signaling also contribute to organogenesis. Local auxin biosynthesis in the SAM mediated by the *YUC* genes contributes to organ formation, as high-order *yuc* mutants do not produce flower buds (Cheng *et al.*, 2006). Similarly, the preferential localization of auxin signaling elements (ARFs and Aux/IAA) in the periphery of the SAM, determined by *in situ* hybridization, also contributes to enhance the auxin effect at the site of auxin maximum (Vernoux *et al.*, 2011).

The phyllotactic pattern controlled only by auxin would be noisy (Besnard *et al.*, 2014). This noise is reduced by a second ‘inhibitory field’ mediated by AHP6, which acts as a negative regulator of cytokinin signaling (Besnard *et al.*, 2014). Gene expression of *AHP6* is induced at sites with an auxin maximum by the auxin response transcription factor ARF5, whereby the protein AHP6 diffuses into surrounding tissues where it is not expressed, acting non-cell autonomously to prevent cytokinin-triggered organ initiation (Fig. 2C). Thus, a new primordium is formed only at the site where the two ‘inhibitory fields’ are lowest, namely the lowest auxin level and the lowest inhibition of cytokinin responsiveness.

### The spatial interplay of plant hormone pathways regulates the plant response to shade

Plants perceive shade or future shade, when the light is reflected by the neighboring plants, due to a reduction of the red/far-red light ratio, which inactivates the photoreceptor PhyB that plays a central role in the shade response (Casal, 2013). In both cases, plants perceive the reduction in ratio as a stress signal that triggers a series of developmental and growth responses to avoid the shade. These responses, collectively known as shade avoidance, include the rapid elongation of the hypocotyl and leaf petioles, a reduction of cotyledon growth, and leaf hyponasty. Shade avoidance involves the coordinated response of various organs, and is achieved by spatial regulation of plant hormone pathways at the metabolic, transport, and signal transduction level. The response is orchestrated by auxin, which further amplifies the signal by regulating other hormones, such as gibberellins, brassinosteroids, and cytokinins.

#### Spatially coordinated auxin activity orchestrates the growth response to shade

Shade is perceived by cotyledons and leaf blades, triggering local auxin biosynthesis in both organs (Procko *et al.*, 2014; Michaud *et al.*, 2017; Pantazopoulou *et al.*, 2017). The induction of auxin biosynthesis is mediated by the bHLH transcription factors PHYTOCHROME INTERACTING FACTOR4 (PIF4), PIF5, and PIF7, which activate several *YUCCA* (*YUC*) genes (Kohnen *et al.*, 2016). Specifically, *YUC2*, *YUC5*, *YUC8*, and *YUC9* genes are induced by shade, are expressed in shade-responsive tissues, and are required for the full response to shade (Müller-Moulé *et al.*, 2016), similar to the *TRYPTOPHAN AMINOTRANSFERASE OF ARABIDOPSIS1* (*TAA1*) gene encoding the enzyme that catalyzes the first step in the pathway (Tao *et al.*, 2008).

**Fig. 3.**
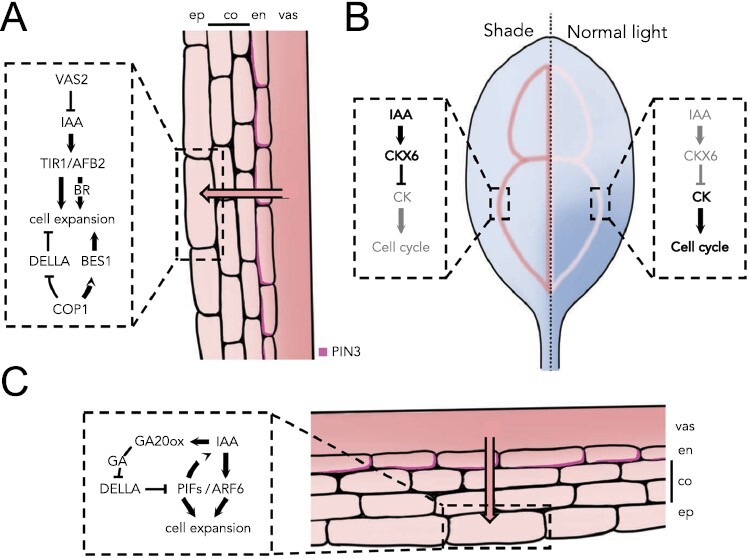
Hormone-mediated spatial regulation of organ growth in response to shade in Arabidopsis. (A) The elongation of the hypocotyl is stimulated by shade through the local action of auxin in the epidermis, which is transported from the cotyledons through the vasculature and translocated thanks to the PIN efflux carriers. (B) Cotyledon growth is arrested under shade conditions by the increase of auxin in the vasculature, which induces the expression of *CKX* that reduces cytokinin levels and thus cell division. (C) Hyponasty is caused by differential petiole elongation in response to shade, which is due to the combined effect of transported auxin and local gibberellin synthesis. Asymmetric hormone accumulation causes preferential elongation of the abaxial side of the organ by stimulating the activity of the transcription factors PIF and ARF. Red, auxin; blue, cytokinins.

Auxins synthesized in cotyledons and leaves are transported to the hypocotyl, as evidenced by the fact that treatment with the auxin transport inhibitor *N*-1-naphthylphthalamic acid (NPA) increases auxin responsiveness in cotyledons and leaves and decreases it in hypocotyls and petioles (Tao *et al.*, 2008; Müller-Moulé *et al.*, 2016; Michaud *et al.*, 2017). Importantly, PIN3 increases its expression and is preferentially localized to the lateral, outer side of the endodermis cells of the hypocotyl in response to shade (Fig. 3A) (Keuskamp *et al.*, 2010). Thus, the asymmetric distribution of PIN3 in the endodermis of the hypocotyl redirects the auxin flow coming basipetally through the vasculature toward the epidermis to promote elongation. Similarly, shade leads to asymmetric accumulation of PIN3 in petioles, where it is more abundant on the abaxial side, promoting asymmetric auxin accumulation and differential growth, leading to hyponasty (Fig. 3B) (Küpers *et al.*, 2023).

Shade also acts at the signal transduction level, since the response in hypocotyls is enhanced by induced expression of auxin receptor genes *TRANSPORT INHIBITOR RESPONSE1* (*TIR1*) and *AUXIN SIGNALING F-BOX2* (*AFB2*) in vascular tissue and by simultaneous reduction of the expression of *miR393A*, which targets the receptors’ mRNA for degradation (Pucciariello *et al.*, 2018).

The epidermis is the main tissue that drives growth (Savaldi-Goldstein *et al.*, 2007) and consequently also plays a major role in the hypocotyl response to shade (Procko *et al.*, 2016). As mentioned above, in response to shade, the auxin flux is directed to the hypocotyl epidermis (Keuskamp *et al.*, 2010), where it accumulates (Pucciariello *et al.*, 2018). In addition to auxin transport, auxin inactivation by conjugation via REVERSAL OF SAV2 (VAS2)/GRETCHEN HAGEN3.17 (GH3.17) also contributes to maintain the right pool of active auxins, presumably in the hypocotyl epidermis (Fig. 3A) (Zheng *et al.*, 2016). Specifically, the expression of *VAS2/GH3.17* decreases in hypocotyls in shade, thus contributing to the increase of active auxins in the epidermis. The relevance of the local action of VAS2/GH3.17 in the hypocotyl is supported by the fact that the enhanced response to shade of *vas2* mutants is not affected by treatments with NPA (Zheng *et al.*, 2016). Consistent with these results, auxin signaling in the epidermis makes a greater contribution to shade-induced hypocotyl elongation than in internal tissues (Procko *et al.*, 2016), suggesting that auxin signaling is spatially gated to promote hypocotyl elongation in shade.

#### The growth response to shade is mediated by the local action of other plant hormones that are dependent on and independent of auxin

Auxins act upstream of other plant hormones to spatially regulate the response to shade. For example, primary leaves slow down their growth rate in shade due to a reduction in cell division starting from the tip of the leaf, a process that is triggered by auxins and mediated by cytokinins (Carabelli *et al.*, 2007). In shade, auxin induces the expression of the cytokinin oxidase gene *CKX6* specifically in the veins of the primary leaf, leading to a reduction in the pool of active cytokinins (Fig. 3C). The leaf size of the *ckx6* mutant does not show any reduction after transfer to shade, suggesting that cytokinin degradation in the vasculature is key to arrest the cell cycle in leaf primordia in response to shade (Carabelli *et al.*, 2007). Similarly, part of the auxin action in the hypocotyl epidermis in response to shade is mediated by brassinosteroids, most probably through auxin-induced brassinosteroid biosynthesis (Fig. 3A) (Procko *et al.*, 2016). For example, exogenous treatment with brassinolide, an active brassinosteroid, is able to partially suppress the hypocotyl growth arrest caused by auxin signaling blockage specifically in the epidermis. In petioles, auxin promotes the synthesis of gibberellins in the abaxial side to enhance cell expansion leading to hyponasty (Fig. 3B) (Küpers *et al.*, 2023). The accumulation of auxin in the lower part of the petiole promotes the expression of the *AtGA20ox1* and *AtGA20ox2* genes, which most probably increases gibberellin content, leading to the degradation of DELLA proteins. Interestingly, this appears to be part of a feedforward loop in which gibberellins help to further increase auxin synthesis in the abaxial petiole.

Not all growth responses to shade are auxin dependent. For example, the E3 ubiquitin ligase CONSTITUTIVELY PHOTOMORPHOGENIC1 (COP1) is involved in destabilizing key signaling elements of the gibberellin and brassinosteroid pathways in response to shade (Fig. 3A) (Blanco-Touriñán *et al.*, 2020; Costigliolo-Rojas *et al.*, 2022). COP1 directly promotes the degradation of DELLA proteins in the hypocotyl epidermis to rapidly promote elongation when the seedling senses shade, a process that precedes gibberellin-induced degradation, which occurs later (Blanco-Touriñán *et al.*, 2020). Interestingly, the effect of COP1 on brassinosteroid signaling is organ dependent. While COP1 directly promotes BRI1-EMS-SUPRESSOR1 (BES1) degradation to slow cotyledon growth rate in the shade, it indirectly promotes BES1 accumulation in the hypocotyl to enhance elongation (Costigliolo-Rojas *et al.*, 2022).

### Spatial plant hormone regulation and root responses to water availability

Plants rely on their root system architecture for strong anchorage and to optimize water and nutrient acquisition. Specifically, water availability acts as an external stimulus for the plant to perceive and respond accordingly at the cellular and morphological level. Uneven distribution of water in the soil triggers two main responses whose mechanisms involve the local action of various plant hormones. One is hydrotropism; that is, the orientation of root growth towards soil moisture (Jaffe *et al.*, 1985). The other is hydropatterning, which is the asymmetric formation of lateral roots on the wet side of the primary root (Bao *et al.*, 2014). A third response, called xerobranching, occurs when the root enters a macropore and loses contact with the soil, and is also spatially controlled by various plant hormones (Orman-Ligeza *et al.*, 2018; Mehra *et al.*, 2022). Lateral root formation is suppressed in the dry region until this region is again in contact with moisture.

#### Local ABA and cytokinin actions regulate hydrotropism

**Fig. 4.**
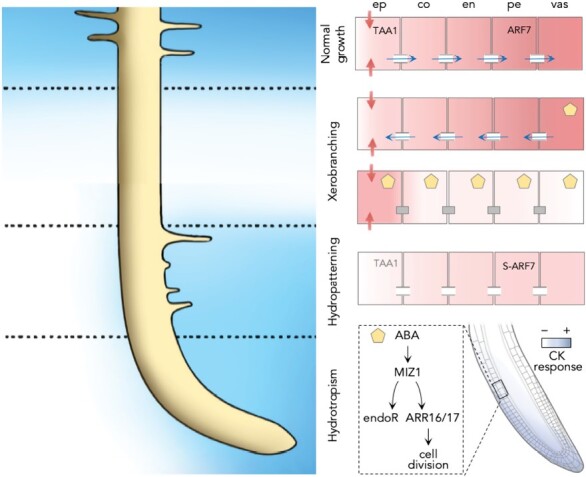
Hormone-mediated shaping of the root in response to water availability. Left, a schematic of a primary root under different situations of water availability (more water, blue; less/no water, white). Right: hypothetical models of the cellular and molecular mechanisms underlying these responses. Red color indicates auxin levels; red arrows, auxin transport; blue arrows, water flow through plasmodesmata; gray boxes, closed plasmodesmata due to ABA signaling; S-ARF7, sumoylated ARF7; ep, epidermis; co, cortex; en, endodermis, pe, pericycle; vas, vasculature; endoR, endoreduplication. For an explanation of mechanisms, please read the main text.

Hydrotropism involves the simultaneous local actions of ABA and cytokinins in different sites. In the region of the root that is exposed to low water potential, ABA signaling in the cortex up-regulates a hydrotropism-specific protein, MIZU-KUSSEI1 (MIZ1) (Fig. 4) (Dietrich *et al.*, 2017). The up-regulation of *MIZ1* expression triggers endoreduplication to increase differential and asymmetric cell expansion of cortical cells (Dietrich *et al.*, 2017). This causes a hydrotropic bending response where the root bends in the direction of a higher water potential. At the same time, the root tip shows a MIZ1-dependent asymmetric cytokinin response, which is probably due to an asymmetric cytokinin distribution that is higher on the lower water potential side of the root (Chang *et al.*, 2019). The high cytokinin activity leads to increased expression of two type A response regulator genes, *ARR16* and *ARR17*, which promote cell division, enhancing a bend toward higher water potential.

#### Hydropatterning is controlled by local action of auxins

Pioneering studies that have coined the term hydropatterning have shown that lateral roots are formed only on the side of the root that is in contact with soil moisture when the root encounters a macropore (Bao *et al.*, 2014). This effect is mediated by enhanced auxin biosynthesis by TAA1 in the epidermis of the wet side and auxin transport by PIN2, PIN3, and PIN7, leading to asymmetric specification of lateral roots in that side (Fig. 4). Specifically, PIN3 is localized to all surfaces of cortex and endodermal cells around lateral root primordia in the wet side. Auxin signaling mainly in the cortex and endodermis of the wet side is also required for the lateral root specification (Bao *et al.*, 2014).

The transcription factor ARF7 promotes lateral root formation (Okushima *et al.*, 2005). Interestingly, differential regulation of ARF7 by sumoylation across the root also contributes to hydropatterning (Fig. 4) (Orosa-Puente *et al.*, 2018). On the dry side of the root, in addition to the lack of auxins, lateral root development is inhibited because ARF7 cannot activate the expression of *LATERAL ORGAN BOUNDARIES DOMAIN16* (*LBD16*) due to SUMOylation (Orosa-Puente *et al.*, 2018). As a result of this modification, IAA3 is recruited to SUMOylated ARF7 to form a repressor complex that prevents activation of *LBD16* and therefore the specification of lateral roots in the dry side. On the wet side, however, non-SUMOylated ARF7 cannot recruit IAA3 and thus activate the expression of *LBD16* (Orosa-Puente *et al.*, 2018). Once the dry region of the root is exposed to moisture again, SUMO is cleaved off from ARF7 by SUMO proteases OVERLY TOLERANT to SALT1 (OTS1) and OTS2, and auxin signaling proceeds (Orosa-Puente *et al.*, 2018).

#### Xerobranching is controlled by local action of ABA and auxins

The auxin- and ABA-dependent molecular mechanism that controls xerobranching has been recently elucidated (Mehra *et al.*, 2022). When the root loses contact with the soil, the flow of auxin and ABA through the plasmodesmata inward and outward, respectively, is especially important during transient water stress (Fig. 4) (Mehra *et al.*, 2022). Under hydrated conditions, inward water flow is maintained and small molecules, including auxins, are mobilized along with symplastic flow through the plasmodesmata from the epidermis to the inner pericycle to initiate lateral root formation. However, when roots lose contact with the soil, a flow of water outward from the phloem is essential to maintain growth. During this process, ABA, which is synthesized in the phloem, migrates outward to promote xerobranching (Mehra *et al.*, 2022). Studies on ABA mutants have suggested that ABA signaling may be particularly important in the cortex and endodermis during this response (Mehra *et al.*, 2022). ABA causes closure of plasmodesmata, which blocks the symplastic inward movement of auxin, preventing it from reaching the pericycle and the formation of lateral roots (Mehra *et al.*, 2022). Once the root reaches the moist soil again, the ABA effect is reduced and auxin can initiate lateral root formation.

## Tools for the study of plant hormone action at the spatial level

The development of plant hormone-specific reporter lines has been critical to determine the spatial distribution of hormone action at the organismal level. Over the last 25 years, we have moved from indirect, synthetic transcriptional reporters to sensors based on FRET (fluorescence resonance energy transfer) that directly detect the plant hormone. The race in the development of plant hormone reporters began with the pioneering work of Professor Tom Guilfoyle’s laboratory with the auxin reporter DR5 (Ulmasov *et al.*, 1997). This reporter was based on a synthetic promoter composed of several repeats of the auxin-responsive element TGTCTC driving the expression of the *GUS* (β-glucuronidase) gene. As mentioned in previous sections, this reporter has been of tremendous help in defining the roles of auxin at the organismal level, for example as a morphogen (Benkova *et al.*, 2003). Similar transcriptional reporters for ethylene (Stepanova *et al.*, 2007), cytokinins (Müller and Sheen, 2008), and ABA (Wu *et al.*, 2018) based on specific *cis*-responsive elements were subsequently developed. These transcriptional reporters mark the sites of active plant hormone signaling, which may or may not coincide with the sites of hormone accumulation.

To more accurately determine the spatial distribution of plant hormone levels, a new generation of reporters based on hormone-specific degrons was developed for auxin and jasmonates. These reporters consist of a fusion of a fluorescent protein with the fragment of a hormonal signaling element that confers hormone-induced degradation to the fusion protein so that the fluorescence intensity is inversely proportional to the amount of plant hormone. The first reporter of this type was the auxin sensor DII–Venus, consisting of the DII degron from the IAA28 protein fused to Venus targeted to the nucleus (Brunoud *et al.*, 2012). This reporter has been further improved and is now semi-quantitative. The new ratiometric auxin reporter, named R2D2, contains, in addition to DII, the auxin-insensitive mDII fused to ntdTomato, both under the control of the same promoter (Liao *et al.*, 2015). Normalization of the DII signal to that of mDII allows a semi-quantitative measurement of auxin. A similar approach was followed to develop a reporter for jasmonic acid, Jas9–Venus, although in this case the normalization was done with histone 2B–red fluorescent protein (H2B–RFP) (Larrieu *et al.*, 2015). A variation on this theme was used to develop the Hormone-Activated Cas9-based Repressors (HACRs) that respond to auxins, gibberellins, or jasmonic acid (Khakhar *et al.*, 2018). HACRs consist of two parts, a fusion protein containing dCAS9, the catalytically inactive Cas9, the plant hormone-targeted degron, and the transcriptional repressor TOPLESS, and a nuclear Venus–luciferase controlled by the *UBIQUITIN10* (*UBQ10*) promoter. The hybrid transcriptional repressor is targeted to the *UBQ10* promoter via Cas9 associated with a guide RNA, resulting in promoter activities proportional to plant hormone levels. The system can be ratiometric by introducing a nuclear tdTomato under the control of a mutant *UBQ10* promoter where the guide RNA cannot bind and, thus, independent of the dCas9 control.

The most recent developments in this field have been the design of sensors based on FRET, which interact directly with the plant hormone and detect it with spatial resolution (Balcerowicz *et al.*, 2021). In contrast to reporters based on plant hormone-specific degrons, where the readout is not directly due to the binding of the plant hormone (i.e. depends on the degradation of the reporter protein), sensors based on FRET respond directly to the binding of the hormone. The sensors for ABA and gibberellins, named ABACUS/ABAleon and GPS1, respectively, consist of a fusion between the plant hormone receptor and a fragment of a signaling element with which the receptor normally interacts during hormone binding (Jones *et al.*, 2014; Waadt *et al.*, 2014; Rizza *et al.*, 2017). This hybrid protein contains at each end a fluorescent protein of a pair capable of FRET. The binding of the plant hormone causes a conformational change that alters the FRET emission ratio, which is used as readout to infer the plant hormone levels. Recently, an auxin sensor based on FRET named AuxSen was developed using a different approach. In this case, the bacterial tryptophan repressor, which suffers a conformational change after binding tryptophan, was used (Herud-Sikimić *et al.*, 2021). The tryptophan-binding pocket of the repressor was redesigned to bind auxin, which is structurally related to tryptophan. The binding of auxin causes a conformational change that alters the FRET emission of the attached fluorescent protein pair, allowing the direct quantification of the hormone.

Traditional quantification of plant hormones usually lacks spatial resolution despite the improved sensitivity of analytical methods. An exception was the analysis of auxin levels in all tissues of the root tip (Petersson *et al.*, 2009). Here, auxin levels were determined in protoplasts isolated from each tissue by cell sorting. The results confirmed the existence of an auxin gradient, as previously suggested by the use of DR5 or DII–Venus reporters, with the highest concentration in the quiescent center, and suggested the existence of local auxin biosynthesis at the root tip, as later shown (Brumos *et al.*, 2018). This approach would be equally suitable to determine levels of other plant hormones, provided that the analytical methods are sensitive enough.

## Perspectives

We currently have a fairly good understanding of a number of plant hormone-regulated processes that occur at specific sites in the plant. The use of reporter lines, especially direct plant hormone sensors, and the detection of plant hormones and precursors in the vascular stream have made it clear how dynamic and spatially regulated plant hormone responses are. All this only shows that we are beginning to expose the complexity of plant hormone effects in time and space. It is expected that the development of improved versions of the existing direct sensors and new sensors for the other plant hormones will help to provide a more complete dynamic and spatial picture of plant hormone action at the organism level.

The recently developed MSI opens up the possibility of quantifying plant hormone levels with spatial resolution (Hansen *et al.*, 2018). The use of matrix-assisted laser desorption ionization (MALDI) greatly reduces the sample size to micrometers, which, combined with the extremely high sensitivity and chemical specificity, makes this technology promising for defining plant hormone cell maps using intact organs or organ sections. In this sense, the improvement of current methods for single-cell metabolomics will probably extend to the quantification of plant hormones (Fujii *et al.*, 2015; de Souza *et al.*, 2020).

In plants, the recently implemented genomic technique of single-cell RNA-sequencing (scRNA-seq) offers a new way to decipher plant hormone action at the spatial level with cellular resolution. ScRNA-seq interrogates the transcriptome of a multitude of individual cells within a given organ, tissue, or pool of cells (Eberwine *et al.*, 2014). It allows not only the definition of the cellular atlas of a specific organ or tissue, but also the study of the response to endogenous and environmental signals at the cellular level (Eberwine *et al.*, 2014; Jean-Baptiste *et al.*, 2019; Kim *et al.*, 2021; Yang *et al.*, 2021; Xia *et al.*, 2022). Two examples illustrate the power of scRNA-seq to determine the spatial effects of plant hormones. This approach has shown that root protoxylem and columella cells are largely insensitive to cytokinins and has enabled the identification of novel tissue-specific cytokinin marker genes (Yang *et al.*, 2021). Time series scRNA-seq analyses of the response to brassinosteroids in roots have identified a role for this hormone in the transition from cell proliferation to cell elongation specifically in the cortex, mediated by two transcription factors not previously associated with brassinosteroids (Nolan *et al.*, 2023). The transcriptional profiling of individual cells not only in response to treatment with a plant hormone but also in metabolic, transport, or signaling mutants will help define the cellular map of plant hormone action in different plant organs.
